# Three-dimensional soft tissue comparison in non-growing patients treated for skeletal Class II Division I malocclusion: mandibular advancement surgery *vs*. Herbst Appliance

**DOI:** 10.1590/1678-7757-2025-0310

**Published:** 2025-11-03

**Authors:** Gökhan ÇOBAN, Taner ÖZTÜRK, Gökhan TÜRKER, Ebru Topcuoğlu HASHIMLI, Yusuf Nuri KABA

**Affiliations:** 1 Erciyes University Department of Orthodontics Faculty of Dentistry Kayseri Turkey Erciyes University, Department of Orthodontics, Faculty of Dentistry, Kayseri, Turkey.; 2 Autonomous researcher Mersin Turkey Autonomous researcher, Mersin, Turkey.; 3 Uludag University Faculty of Dentistry Department of Oral and Maxillofacial Surgery Bursa Turkey Uludag University, Faculty of Dentistry, Department of Oral and Maxillofacial Surgery, Bursa, Turkey.

**Keywords:** Orthognathic surgery, Anthropometric soft tissue landmarks, Herbst appliance, Dentofacial orthopedics, Stereophotogrammetry

## Abstract

**Objective:**

This study used three-dimensional (3D) stereophotogrammetry to compare changes in facial soft tissues of patients with skeletal class II malocclusion with mandibular retrognathia treated with orthognathic surgery (OS) or Herbst appliance (HA).

**Methodology:**

This retrospective study included 15 adults treated with bilateral sagittal split osteotomy without genioplasty (OS) and 16 adults treated with HA. The patients were treated successfully, with class I occlusal relationships with normal overjet and overbite. Three-dimensional stereophotogrammetric records acquired in habitual occlusion from before and after fixed orthodontic treatment were analyzed. Lateral cephalometric radiographs were used to evaluate skeletal parameters and sagittal oropharyngeal airway length.

**Results:**

In HA-group, mandibular corpus length, anterior facial height, posterior facial height, Bº, vertical angle, and mentolabial angle increased; lower facial width, mandibular angle, mandibular convexity angle, ANBº, and Aº decreased. In OS-group, mandibular length, mandibular corpus length, and facial convexity angle increased significantly compared with those in HA-group; posterior facial height, Bº, vertical angle, and mentolabial angle also increased, but mandibular angle and ANBº decreased significantly. Only a significant difference in coordinate changes was observed for the pogonion in the sagittal direction. According to the cephalometric analysis, SNBº and Pg-NA perpendicular measurements increased significantly in the OS group compared with the HA group. In both groups, sagittal oropharyngeal airway length increased significantly post-treatment, with no significant intergroup difference.

**Conclusion:**

In patients treated with OS, skeletal advancement resulted in greater increases in mandibular and corpus lengths, along with forward positioning of the pogonion. Based on 3D soft tissue and cephalometric comparisons, the Herbst appliance seems to be a viable non-surgical alternative for young adults with moderate Class II malocclusion. Both treatment modalities also contributed to significant improvements in sagittal oropharyngeal airway length.

## Introduction

One-fourth of the population have class II malocclusions, which predominantly result from genetic and environmental factors (i.e., parafunctional behaviors, such as persistent finger and tongue sucking habits, lip trap, or early loss of deciduous teeth) that can be observed at variable rates over time.^1,2^

Class II, division I malocclusions are the most common form of malocclusion^3^ and are often characterized by proclined maxillary incisors with or without a relatively narrow maxilla, an increased overjet, incomplete lip closure, and mandibular skeletal retrusion, which is the most common diagnostic result.^1,4,5^

The backward positioning of the tongue, due to mandibular retrusion, may cause a narrowing of the pharyngeal airway and breathing difficulties.^6^ Moreover, retrognathic mandible can cause sleep-disordered breathing, which ranges from snoring to stroke.^7^ Moreover, maxillary incisor trauma increases in class II malocclusions, and oral health-related quality of life decreases.^8-10^

The treatment of skeletal class II malocclusion, which is observed with all those characteristics, is important not only to address chewing and biting but also to improve functions such as breathing and speaking as well as aesthetic, psychosocial, and self-image development.^11^

Three basic approaches have been proposed in the treatment of skeletal malocclusion: growth modification, camouflage, and orthognathic surgery.^12^ Although growth modification is the first option for individuals who are still growing and developing, camouflage treatment can be also applied. Late adolescents and adults no longer have significant growth, so the treatment options are camouflage and orthognathic surgery.^13^

However, effective correction can also be achieved with the Herbst appliance (HA), the most frequently used fixed bite jumping orthopedic device,^14^ in class II malocclusions, even in adults, and it may be an alternative to extraction treatments and orthognathic surgery.^15^ The HA keeps the mandible in a constantly forced protruded position when the mouth is open or closed. Because of this mechanical stress, different degrees of changes can be seen in the mandible (and its position^12^), muscle function, and temporomandibular joint (condyle-glenoid fossa) structures, which, combined with dentoalveolar effects, depend on age and maturity stage.^3,16^ Additionally, the HA stimulates condylar growth and remodels the glenoid fossa in children and adults.^17^ For all these reasons, the HA can be considered a fixed functional appliance.^18^ Evidence indicates that growth-adapting treatment with the HA is possible even after 20 years and that it can replace or be an alternative to surgery, especially in treatment for mild skeletal class II^15^, and its stability was as high as that of orthognathic surgery in a three-year long-term follow-up.^19^

Various types of HAs have been used, depending on the method of attachment to the teeth. These are banded HA, cast splints, acrylic splints, and stainless-steel crowns.^20^ The cast splint and banded versions are cemented to the teeth whereas the acrylic splint version requires an interocclusal acrylic layer, and it can design to be complete or partially removable.^21^ The stainless-steel cast splint HA has several advantages over other types: it fits perfectly, it is rigid and hygienic, the chair time is short, and problems such as band or crown breaks are less common.^18^

Combined orthodontic and orthognathic surgery (OS) is a well-known method that has been used for about 70 years; It has a high success rate in resolving the underlying skeletal discrepancy in skeletal class II malocclusions, correcting function, and improving soft tissues by reducing facial convexity, thus improving aesthetics.^22^ The most common mandibular surgical procedure is mandibular bilateral sagittal split osteotomy (BSSO).^23^

The stereophotogrammetry method, which was introduced to dentistry in the 1940s and is frequently used in 3D imaging of the face^24^, is fast, easy, and non-invasive and enables the evaluation of facial soft tissues without radiation.^25^ This provides comprehensive 3D visualization and precise assessment of facial structures, making it particularly suitable for evaluating and comparing morphological changes in orthodontic and surgical treatments.^26^

Although studies have compared the treatment effectiveness and outcomes of dentofacial orthopedics and OS,^3,15,27^ no studies have compared the treatment effectiveness of these two methods using 3D images. Therefore, this study compared the changes in the soft tissues of patients (whose growth was completed) who were treated with the cast splint stainless-steel HA and mandibular advancement with BSSO (without genioplasty) using the stereophotogrammetry method.

## Methodology

This was a retrospective, cross-sectional, and observational study involving a 3D outcome analysis of two groups of patients, which was approved by the Erciyes University Ethics Committee (Approval Code: 2023/695). The study was conducted ethically in accordance with the guidelines of the World Health Organization’s Declaration of Helsinki.

### Sample size calculation

The G*Power (v. 3.1.9.7, Franz Faul, Universität Kiel, Germany) software was used to determine the sample size. The results reported by Ruf and Pancherz (N–Sn–Pog facial convexity angle) were used for power analysis, and the following parameters were applied: 1-alpha=0.05, 90% power, and d=0.812. The calculation indicated that at least 15 individuals should be included in each group.^15^

### Patients

The study population comprised 15 adults (11 women and four men, mean age: 19.9±2.43 years, range: 17.2–25 years) treated with OS and 16 adults (ten women and six men, mean age: 16.01±0.78 years, range: 15.1–17.1 years) treated with the HA. The patients were recruited between June 2020 and January 2023 and were treated at the Departments of Orthodontics and Oral and Maxillofacial Surgery, Faculty of Dentistry, Erciyes University.

The following patients were included:

- Patients who had skeletal class II, division 1 malocclusion and were treated without extraction (except for the third molars) (ANB° > 4°).
- Patients who had normal vertical growth pattern (30° < SN/GoGn < 35°).
- Patients who had appropriate 3D stereophotogrammetric recordings at the beginning and end of treatment, attended their appointments regularly, had complete follow-up, and did not report any incompatibilities, such as device breakage or usage errors.
- Patients who were treated with HA and OS and had class I occlusions with normal overjet and overbite at the end of treatment.^15^

Patients who had syndromes, congenital anomalies, craniofacial deformities, excessive vertical growth patterns, missing teeth, an overjet of less than 6 mm, severe facial asymmetry, or severe maxillary transverse deficiency, along with those who had previously received orthodontic treatment, and those whose growth and development was not complete (according to hand–wrist radiography evaluation) were excluded from the study.

Skeletal maturity of the patients was classified as described by Ruf and Pancherz^28^ according to hand and wrist roentgens, and all patients were found to be in the R-J stage. Accordingly, all patients in both groups were evaluated as young adults and adults.

### Herbst appliance group

**Figure 1:**
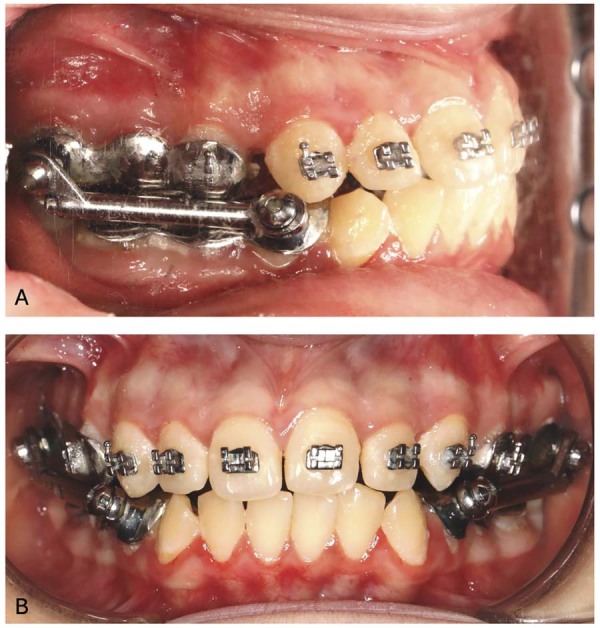
Intraoral images (A-Profile view, B-Frontal view) of the appliance applied in the Herbst appliance group.

The HA type used in this study was a stainless-steel cast splint with a multibracket system. In this type, cast crowns cover the maxillary and mandibular molars and premolar teeth, and a lingual arch connects the mandibular part. A 10 mm hyrax screw (Dentaurum, Pforzheim, Germany) was also used to bring the maxillary parts together and provide expansion. This was activated via a semi-rapid protocol (two turns on the first three days, one turn on the following four days, one turn on every following two days) until adequate expansion was achieved. A Dentaurum type 1 telescopic system (Herbst Set I; Dentaurum, Ispringen, Germany) was used.^16^ Guide tubes were soldered to the steel crowns on the upper second molar teeth, and slide pins were soldered to the steel crowns on the lower first premolar teeth. The construction process of this device was performed by experienced technicians in a laboratory on plaster models taken from the patient. The HA was cemented to the teeth with 3M ESPE glass ionomer cement (Ketac-Cem; 3M ESPE, Seefeld, Germany). Moreover, because a multibracket HA was used, premolar brackets were placed on the stainless-steel crowns on the upper first premolar teeth, and Roth prescription 0.018” brackets (Mini Master Series, American Orthodontics, Sheboygan, WI, USA) were placed on the upper anterior teeth (Figure 1). Orthodontic leveling and alignment was performed up to 0.016” × 0.022” stainless-steel (SS) wire. Sagittal activation was performed in the first appointment in which the HA was applied, and activations after the first 4-5 mm of forward movement of the mandible were conducted by adding shims until normal overjet was achieved.^29^ After the HA was removed, all remaining teeth were bonded, and 3D stereophotogrammetric recordings were taken before the HA was applied (T0) and immediately after debonding (T1). As a result of fixed functional and subsequent orthodontic treatment, class I occlusion was achieved in molar and canine relations, normal overjet values, and good interdigitation, then all devices were removed.

### Orthognathic surgery group

This group included patients who underwent mandibular advancement alone with BSSO without genioplasty. BSSO was performed by the same experienced surgical team according to Hunsuck’s modification.^15^ The same surgical and anesthesia techniques were performed for all patients.

### Pre- and post-surgical orthodontic procedure

For all patients, 0.018” slot Roth prescription brackets (Mini Master Series, American Orthodontics, Sheboygan, WI, USA) with straight wire technique were used. Treatment was initiated with 0.012” or 0.014” Nickel-titanium (NiTi) wires for alignment and leveling, and 0.016”, 0.016”x0.022” NiTi, and 0.016”x0.022” Stainless Steel (SS) arch-wires were used for incisor decompensation and arch coordination, respectively. Patients were seen for four to six weeks. In all cases, a final surgical splint produced conventionally or with a virtual surgery planning software (Nemo FAB, Nemotec, Leganés, Madrid, Spain) was used to ensure the planned occlusion. The pre-surgical orthodontic treatment phase was ten to 12 months. Surgical arch wires with crimpable hooks were placed in the mouth one day before the surgery and replaced with 0.016”x0.022” NiTi arch-wire one month after the surgery.

Post-surgical orthodontics is necessary to perform decompensation or minor dental movements that cannot be performed because of the iatrogenic origin or disto-occlusion or mastication function and force.^30^ The post-surgical orthodontic treatment is focused on finishing and detailing, so after achieving Class I occlusion, normal overjet values, and good interdigitation, the treatment was finished, and the orthodontic devices were removed.

Three-dimensional stereophotogrammetric recordings were taken immediately before surgery (T0) and immediately after debonding (T1), which was performed at least six months after surgery to ensure complete resolution of edema and soft tissue adaptation to BSSO.

### Surgical procedure

Surgical procedures were performed according to the procedure described below.^31^ The same team performed the operations under general anesthesia. Bilateral inferior alveolar nerve block and local infiltrating anesthesia were applied. A mucosal incision was made with the electrocautery, and the mucoperiosteal flap was lifted to expose the medial and the lateral surface of the ramus and the lower border. Medial horizontal osteotomy was performed with ultrasonic devices just above the lingula and extending behind the lingula. Oblique osteotomy was performed starting from the anterior of the medial osteotomy to the mesial part of the second molar tooth. Vertical osteotomy was extended starting from the anterior border of the oblique osteotomy to the lingual surface of the lower border. Osteotomy lines related to chisel, and the split was performed with a border separator and bone spreader. The mandible was moved to the new position with the orthodontic surgical splint. The proximal and distal segments were fixed with mini plates (KLS Martin, Tuttlingen, Germany) and mini screws (KLS Martin, Tuttlingen, Germany). The mucosa was sutured with 4-0 Vicryl. The patient was extubated and transferred to the postoperative care unit.

### Three-dimensional stereophotogrammetric records

**Figure 2:**
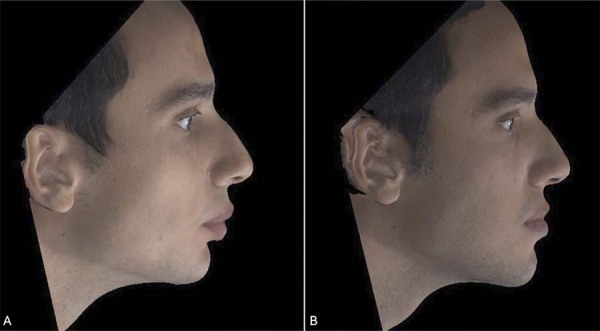
The 3dMD images showing the three-dimensional view before (A) and after treatment (B) of a patient who underwent mandibular advancement surgery.

**Figure 3:**
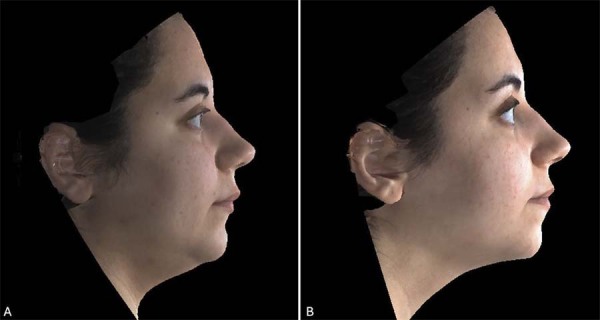
The 3dMD images showing the three-dimensional view before (A) and after treatment (B) of a patient who underwent Herbst appliance therapy.

Three-dimensional stereophotogrammetric records of all patients of this study were obtained with a 3dMDface™ (3dMD Ltd, Atlanta, GA, USA) imaging system in a private room in the orthodontics department. Prior to photography, patients were instructed to remove their glasses and any jewelry, and men were required to shave their beards and/or mustaches. Images were taken with the teeth in slight occlusion, eyes open, and lips relaxed and together (Figure 2 and Figure 3). Care was taken to avoid any distortions or artifacts in the image recordings.

**Figure 4:**
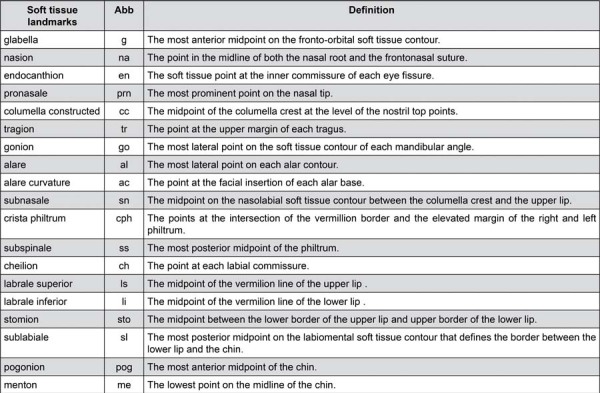
Definitions of soft tissue landmarks and reference planes.

Abb: Abbreviations

### Outcomes

The primary outcome was the difference in facial soft tissue changes—including the mandibular area (pogonion, gonion, and lower lip) as well as other regions, such as the upper lip, nose, and maxilla—between the HA and OS groups, as measured by 3D facial images taken at T0 and T1. Secondary outcomes were skeletal and dental changes, along with pharyngeal airway dimension—specifically the minimum posterior airway space (PASmin)—assessed via lateral cephalometric radiographs.

Images taken at T0 and T1 were evaluated with 3dMDvultus™ (3dMD Ltd, Atlanta, GA, USA) software. These images were manually aligned in the same software program and automatically registered by marking the broad surface of the forehead, the surface from the root of the nose to the hump of the nose, and the lateral parts of the bilateral Ex.^32^ The root mean square (RMS) value calculated for the acceptability of registration was below the recommended value of 0.5^33^ (RMS: 0.09–0.49, mean value: 0.30±0.11).

**Figure 5:**
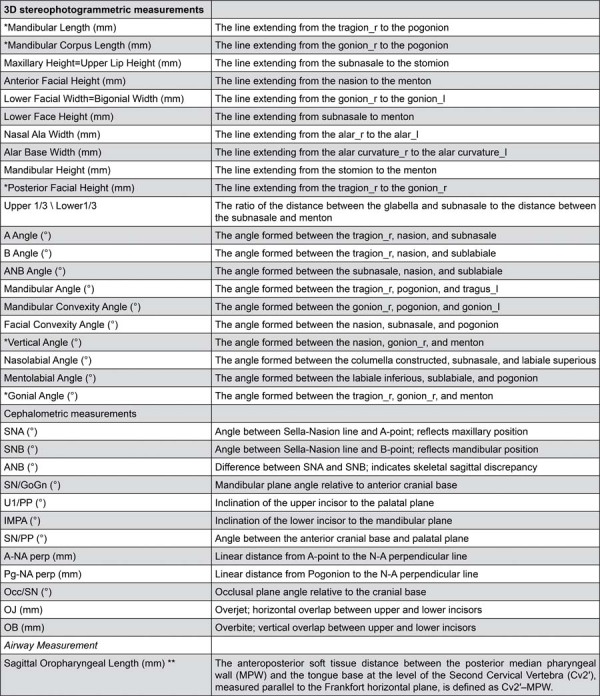
Measurements and their explanations.

Figures 4 and 5 show, respectively, the definitions and abbreviations of the anthropometric landmarks^34^ and the measurements used in the study. For bilateral points, the letter ‘r’ is added on the right, and ‘l’ is added on the left. After the landmarking process of the T0 images was completed, all points were projected onto the superimposed T1 image. The points that were not affected by the surgery (G, N, En_r, and En_l) were left as they were; All other points were repositioned on the T1 images, and the 3D changes (in the horizontal (x), vertical (y), and sagittal (z) directions) of the points at T0 and T1 were compared.

The measurements marked with an asterisk were measured separately for the right and left sides for all patients, and no statistically significant differences were detected between the measurements on either side. Therefore, right-side measurements are provided.

The changes in the 17 anthropometric landmark coordinate changes and the then linear, ten angular, and one rational measurements (Figure 5, Table 1 and 2) were evaluated. Additionally, eight angular and four linear cephalometric parameters were analyzed, along with one linear airway measurement, to provide a comprehensive assessment of skeletal and functional changes (Table 5).

Sagittal Oropharyngeal Length (mm) ** The anteroposterior soft tissue distance between the posterior median pharyngeal wall (MPW) and the tongue base at the level of the Second Cervical Vertebra (Cv2′), measured parallel to the Frankfort horizontal plane, is defined as Cv2′–MPW.

* Since there is no statistical difference between the measurements evaluated separately for the right and left sides, right side measurements are given for the relevant measurements.

** Reference 4

**Table 1:** Pre-treatment values of the Herbst Appliance (HA) and Orthognathic Surgery (OS) Patients.

Measurements	HA	OS	P values*
Mandibular Length (mm)	131.46±7.24	129.58±7.56	0.483
Mandibular Corpus Length (mm)	98.13±7.0	95.21±6.50	0.240
Maxillary Height=Upper Lip Height (mm)	21.17±1.92	21.25±2.92	0.916
Anterior Facial Height (mm)	115.99±5.27	116.40±10.55	0.892
Lower Facial Width=Bigonial Width (mm)	114.18±12.26	110.31±8.73	0.319
Lower Face Height (mm)	68.76±5.56	68.40±9.10	0.895
Nasal Ala Width (mm)	34.59±4.43	32.68±3.51	0.195
Alar Base Width (mm)	28.48±3.96	27.61±2.52	0.477
Mandibular Height (mm)	48.32±4.49	48.06±8.78	0.921
Posterior Facial Height (mm)	55.65±19.94	53.04±8.81	0.646
Upper 1/3 \ Lower1/3	0.98±0.11	0.99±0.11	0.758
A Angle (°)	80.79±2.15	80.18±3.59	0.571
B Angle (°)	72.88±2.14	71.47±3.45	0.179
ANB Angle (°)	13.38±2.99	13.08±1.69	0.726
Mandibular Angle (°)	66.95±2.57	68.30±3.50	0.236
Mandibular Convexity Angle (°)	71.23±4.65	71.27±5.92	0.981
Facial Convexity Angle (°)	155.33±7.65	155.32±4.74	0.992
Vertical Angle (°)	62.99±2.68	62.93±5.80	0.973
Nasolabial Angle (°)	118.52±7.95	112.02±10.97	0.068
Mentolabial Angle (°)	114.52±18.69	120.93±17.22	0.329
Gonial Angle (°)	135.27±5.01	134.04±6.21	0.548

**Table 5:** Comparison of cephalometric changes with treatment in the Herbst Appliance (HA) and Orthognathic Surgery (OS) Patients.

	HA			OS			Comparisons of T0	Differences with Treatment		
	T0	T1	P values*	T0	T1	P values*	P values**	HA	OS	P Values**
SNAº	81.47±3.73	81.41±3.72	0.780	80.31±1.67	80.56±1.66	0.373	0.414	-0.06±0.58	0.24±0.78	0.366
SNBº	75.29±3.97	76.99±3.91	**0.002**	72.48±2.53	75.77±2.68	**<0.001**	0.096	1.7±1.14	3.29±1.62	**0.030**
ANBº	6.19±2.33	4.42±2.55	**<0.001**	7.82±1.16	4.77±2.24	**<0.001**	0.084	-1.77±0.69	-3.06±1.8	0.062
SN/GoGnº	30.56±8.37	31.57±7.65	**0.032**	34.1±7.83	34.77±5.69	0.562	0.368	1.01±1.17	0.67±3.31	0.772
U1/PPº	109.68±9.26	113.22±6.84	0.234	109.07±8.96	112.04±6.81	0.386	0.889	3.54±8.25	2.98±9.74	0.896
IMPAº	94.04±8.07	99.88±6.77	**0.001**	94.22±9.96	95.92±8.59	0.357	0.967	5.83±3.49	1.7±5.22	0.066
SN/PPº	9.62±3.27	9.6±3	0.960	8.17±4.27	6.48±3.02	0.064	0.429	-0.02±1.29	-1.69±2.36	0.087
A-NA perp (mm)	-0.68±3.82	-0.76±3.78	**0.669**	-2.21±1.8	-1.86±2.06	0.290	0.299	-0.08±0.53	0.36±0.94	0.250
Pg-NA perp (mm)	-9.49±6.68	-7.3±6.31	0.006	-15.14±5.69	-9.62±5.66	**<0.001**	0.072	2.19±1.79	5.52±2.24	**0.003**
Occ/SNº	15.58±4.6	17.33±4.41	0.133	14.87±3.86	16.66±3.6	0.182	0.727	1.76±3.15	1.79±3.67	0.983
OJ (mm)	6.53±1.45	3.08±2.48	**0.003**	8.81±2.89	3.68±1.14	**0.002**	0.057	-3.46±2.51	-5.13±3.31	0.243
OB (mm)	5.07±2.05	1.86±1.56	**<0.001**	4.99±3.27	2.16±1.27	**0.010**	0.953	-3.21±1.39	-2.83±2.53	0.700
Airway (mm)	4.31±1.71	5.64±1.84	**<0.001**	4.27±1.34	5.47±1.34	**<0.001**	0.952	1.33±0.58	1.2±0.71	0.683

PP: palatal plane, Occ: Occlusal line, OJ: overjet, OB: overbite. Data was given as mean ± standard deviation. T0: Pre-treatment. T1: Post-treatment. * Results of Paired Samples t-test. ** Results of the Independent samples t-test. Statistically significant value: p<0.05.

### Statistical analysis

The data obtained in the study were recorded using Microsoft Office Excel (Microsoft Office 365, Microsoft, Redmond, Washington, USA) software and statistically analyzed with Statistical Package of the Social Sciences (v. 24.0, IBM Inc., Armonk, NY, USA) software. The Shapiro-Wilk test was used for normality analysis, and Levene’s test was used for homogeneity analysis. All data were normally and homogeneously distributed; therefore, comparisons before and after treatment were made using paired samples t-tests, and comparisons between groups were made using independent samples t-tests. Data are shown as the mean and standard deviation, and statistical significance was defined as p<0.05.

### Method error

The same researcher (ETH) re-analyzed the data from ten randomly selected patients one month after the first measurement. The intraclass correlation coefficient was used to examine the reliability between the first and second measurements. Cronbach’s alpha was 0.978 (range: 0.962-0.989), indicating sufficient reliability.

## Results

All subjects in both treatment groups were treated to a Class I dental arch relationship. The HA treatment time was seven to wight months, and the total treatment time was 19.8 months. The average body mass index (BMI) of the patients in the HA group was 20.48 at the pre- and 20.8 at post-treatment. The total treatment time was 24.2 months in the OS group, with a post-surgical phase lasting approximately six months. The mean BMI in this group was 21.9 at the pre- and 21.2 at post-treatment.

Table 1 shows the comparison of pre-treatment values between groups . None of the parameters differed between the groups at the beginning of treatment.

Table 2 shows the between-group comparison of the changes that occurred after treatment. In the HA group, the lower facial width (p=0.008), mandibular angle (p=0.027), mandibular convexity angle (p=0.006), ANB angle (p<0.001), and A angle (p=0.039) decreased significantly after treatment, whereas the mandibular corpus length (p<0.001), anterior facial height (p=0.019), posterior facial height (p=0.013), B angle (p=0.001), vertical angle (p=0.028), and mentolabial angle (p<0.001) increased significantly after treatment.

**Table 2:** Pre- and post-treatment comparisons of the Herbst Appliance (HA) and Orthognathic Surgery (OS) Patients.

Measurements	HA			OS		
	T0	T1	P values*	T0	T1	P values*
Mandibular Length (mm)	131.46±7.24	132.38±7.44	0.105	129.58±7.56	133.65±7.32	**<0.001**
Mandibular Corpus Length (mm)	98.13±7.0	100.74±6.77	**<0.001**	95.21±6.50	100.16±6.88	**<0.001**
Maxillary Height=Upper Lip Height (mm)	21.17±1.92	21.33±2.14	0.638	21.25±2.92	20.86±2.52	0.246
Anterior Facial Height (mm)	115.99±5.27	117.39±5.86	**0.019**	116.40±10.55	118.08±8.62	0.136
Lower Facial Width=Bigonial Width (mm)	114.18±12.26	110.33±10.97	**0.008**	110.31±8.73	109.46±9.06	0.440
Lower Face Height (mm)	68.76±5.56	69.52±6.06	0.348	68.40±9.10	69.35±6.87	0.476
Nasal Ala Width (mm)	34.59±4.43	34.50±4.33	0.497	32.68±3.51	32.55±3.34	0.406
Alar Base Width (mm)	28.48±3.96	28.85±3.96	0.067	27.61±2.52	27.49±2.39	0.338
Mandibular Height (mm)	48.32±4.49	48.83±5.46	0.493	48.06±8.78	49.58±7.32	0.268
Posterior Facial Height (mm)	55.65±19.94	58.88±19.81	**0.013**	53.04±8.81	55.93±9.18	**<0.001**
Upper 1/3 \ Lower1/3	0.98±0.11	0.97±0.10	0.422	0.99±0.11	0.98±0.10	0.403
A Angle (°)	80.79±2.15	79.88±2.44	**0.039**	80.18±3.59	79.86±3.72	0.381
B Angle (°)	72.88±2.14	74.17±2.24	**0.001**	71.47±3.45	73.72±3.95	**<0.001**
ANB Angle (°)	13.38±2.99	11.53±2.89	**<0.001**	13.08±1.69	10.14±2.73	**<0.001**
Mandibular Angle (°)	66.95±2.57	65.96±1.53	**0.027**	68.30±3.50	66.25±3.38	**<0.001**
Mandibular Convexity Angle (°)	71.23±4.65	69.71±3.41	**0.006**	71.27±5.92	70.14±6.04	0.132
Facial Convexity Angle (°)	155.33±7.65	156.84±6.62	0.060	155.32±4.74	160.41±5.93	**<0.001**
Vertical Angle (°)	62.99±2.68	64.10±3.34	**0.028**	62.93±5.80	64.34±5.11	**0.007**
Nasolabial Angle (°)	118.52±7.95	119.90±7.95	0.327	112.02±10.97	111.94±13.53	0.964
Mentolabial Angle (°)	114.52±18.69	132.23±23.67	**<0.001**	120.93±17.22	134.33±14.71	**0.001**
Gonial Angle (°)	135.27±5.01	134.77±3.93	0.723	134.04±6.21	134.82±6.32	0.318

Data was given mean ± standard deviation. T0: Pre-treatment. T1: Post-treatment. * Results of Paired Samples -t- test. Statistically significant value: p<0.05.

In the OS group, mandibular length (p<0.001), mandibular corpus length (p<0.001), facial convexity angle (p<0.001), posterior facial height (p<0.001), B angle (p<0.001), vertical angle (p=0.007), and mentolabial angle (p=0.001) increased significantly after treatment, whereas the mandibular angle (p<0.001) and ANB angle (p<0.001) decreased significantly after treatment.

Table 3 shows the intergroup comparison of changes after treatment. In the OS group, the mandibular length (OS: 4.07±2.54; HA: 0.92±2.12; p=0.001), mandibular corpus length (OS: 4.94±2.51; HA: 2.63±1.46; p=0.005), and facial convexity angle (OS: 5.10±3.29; HA: 1.51±2.96; p=0.003) increased significantly, and the increase was greater than that in the HA group. Moreover, a significant difference was observed in alar base width between the HA group and the OS group (HA: 0.37±0.75; OS: −0.12+0.47; p=0.039).

**Table 3:** Comparison of treatment changes in the Herbst Appliance (HA) and Orthognathic Surgery (OS) Patients.

Measurements	HA	OS	P values*
Overjet Change (mm)	7.41±2.48	7.90±2.97	0.638
Mandibular Length (mm)	0.92±2.12	4.07±2.54	**0.001**
Mandibular Corpus Length (mm)	2.63±1.46	4.94±2.51	**0.005**
Maxillary Height=Upper Lip Height (mm)	0.17±1.40	-0.40±1.26	0.251
Anterior Facial Height (mm)	1.40±2.13	1.68±4.12	0.811
Lower Facial Width=Bigonial Width (mm)	-3.85±4.99	-0.85±4.16	0.081
Lower Face Height (mm)	0.76±3.12	0.95±5.00	0.899
Nasal Ala Width (mm)	-0.09±0.51	-0.13±0.56	0.853
Alar Base Width (mm)	0.37±0.75	-0.12±0.47	**0.039**
Mandibular Height (mm)	0.51±2.89	1.52±5.12	0.497
Posterior Facial Height (mm)	3.24±4.60	2.89±2.37	0.796
Upper 1/3 \ Lower1/3	-0.01±0.05	-0.02±0.07	0.760
A Angle (°)	-0.91±1.60	-0.31±1.35	0.277
B Angle (°)	1.29±1.31	2.25±1.71	0.090
ANB Angle (°)	-1.85±1.00	-2.93±2.13	0.079
Mandibular Angle (°)	-0.99±1.63	-2.05±1.69	0.086
Mandibular Convexity Angle (°)	-1.51±1.92	-1.13±2.73	0.652
Facial Convexity Angle (°)	1.51±2.96	5.10±3.29	**0.003**
Vertical Angle (°)	1.11±1.82	1.41±2.82	0.727
Nasolabial Angle (°)	1.38±5.43	-0.07±5.97	0.485
Mentolabial Angle (°)	17.72±13.51	13.40±13.14	0.375
Gonial Angle (°)	-0.50±2.76	0.78±2.92	0.220

Data was given mean ± standard deviation. * Results of Independent Samples -t- test. Statistically significant value: p<0.05.

Table 4 shows the changes in the coordinates of anthropometric landmarks after treatment and the comparison of these changes between groups. The only point with a significant change in coordinates between groups was the pogonion; in the OS group, this point moved significantly more forward (3.82±2.64; p=0.001) than the same point in the HA group (PG_z: 0.78±2.13).

**Table 4:** Comparison of 3D coordination changes of anthropometric landmarks in the Herbst Appliance (HA) and Orthognathic Surgery (OS) Patients.

Measurements	HA	OS	P values*
G_z	-0.16±0.69	-0.02±0.34	0.502
N_z	-0.25±0.66	-0.13±0.48	0.563
EN_R_z	-0.34±0.68	0.28±0.61	0.283
EN_L_z	-0.19±0.99	0.15±0.44	0.230
AC_R_x	-0.03±0.3	0.12±0.17	0.106
AC_R_z	0.31±1.2	-0.4±1.15	0.103
AC_L_x	0.25±0.6	-0.04±0.28	0.093
AC_L_z	0,57+1,38	-0.07±0.84	0.432
AL_R_x	0.19±0.49	0.06±0.26	0.344
AL_R_z	-0.21±1.09	-0.2±1.13	0.962
AL_L_x	0.05±0.4	-0.08±0.39	0.372
AL_L_z	-0.53±2.28	-0.12±1.19	0.540
Go_R_x	2.21±3.18	0.26±2.17	0.056
Go_R_y	-2.95±3.87	-2.23±2.1	0.523
Go_R_z	0.98±1.92	2.58±2.88	0.078
Go_L_x	-1.64±2.31	-0.6±2.36	0.225
Go_L_y	-2.96±3.87	-2.38±2.12	0.608
Go_L_z	1.09±1.98	2.86±3.48	0.089
CH_R_y	-0.45±1.14	-0.1±1.22	0.420
CH_R_z	0.11±2.51	1.09±2.17	0.258
CH_L_y	-0.31±1.26	-0.07±1.03	0.563
CH_L_z	0.19±2.69	0.73±2.03	0.537
CPH_R_y	0.07±0.28	0.03±0.52	0.807
CPH_R_z	-0.87±1.81	-0.43±1.6	0.473
CPH_L_y	0.12±0.5	-0.08±1.04	0.479
CPH_L_z	-0.84±2.03	-0.42±1.63	0.535
PRN_z	-0.28±0.89	-0.19±0.92	0.790
SN_z	-0.1±1.49	-0.21±0.95	0.810
CC_z	0.34±1.37	0.01±0.62	0.399
SS_y	-0.32±0.68	-0.05±0.38	0.075
SS_z	-0.59±1.87	-0.52±1.93	0.928
LS_y	0.01±0.88	0.33±1.41	0.448
LS_z	-0.69±2.08	-0.38±1.95	0.667
STO_y	-0.38±1.21	0.17±0.93	0.168
STO_z	0.5±2.4	1.46±3.17	0.346
Li_y	-0.46±1.45	-0.19±2.15	0.689
Li_z	1.29±2.28	2.35±3	0.274
SL_y	-2.19±2.35	-1.89±2.08	0.703
SL_z	2.75±2	3.92±3.39	0.245
PG_y	-1.86±3.41	-2.05±2.03	0.855
PG_z	0.78±2.13	3.82±2.64	**0.001**

Data was given mean ± standard deviation. * Results of Independent Samples -t- test. Statistically significant value: p<0.05.

x-axis (horizontal), left (no sign), right (minus sign); y-axis (vertical), superior (no sign), inferior (minus sign); z-axis (sagittal), anterior (no sign), posterior (minus sign).

Parameters are defined in Figure 4

All measurements are given in mm.

Table 5 shows cephalometric comparisons of pre- and post-treatment changes. No statistically significant differences were observed between the HA and OS groups in any cephalometric parameter (p>0.05) at pre-treatment (T0). The HA group showed significant increases in SNB angle (p=0.002), IMPA (p=0.001), SN/GoGn angle (p=0.032), Pg-NA perpendicular distance (p=0.006), and airway dimension (p<0.001) at post-treatment, t. Additionally, significant reductions were noted in ANB angle (p<0.001), overjet (p=0.003), and overbite (p<0.001). SNB angle (p<0.001), Pg-NA perpendicular distance (p<0.001), SN/GoGn angle (p=0.007), and airway dimension (p<0.001) increased significantly after treatment in the OS group. ANB angle (p<0.001), overjet (p=0.002), and overbite (p=0.010) also demonstrated statistically significant reductions. When the magnitude of change was compared between the groups, the OS group showed a significantly greater increase in SNB angle (p=0.030) and Pg-NA perpendicular distance (p=0.003). No significant intergroup differences were found for the change in overjet, overbite, ANB angle, or other variables (*p*>0.05).

## Discussion

In this study, the effects of OS and one of its most common alternatives—the HA (a fixed functional device)^35^—on soft tissues were compared via 3D stereophotogrammetric recordings in patients with skeletal class II malocclusion with mandibular deficiency. All participants had completed growth and development, as determined by hand and wrist X-rays.

The choice of treatment for adult patients with moderate skeletal class II malocclusion varies depending on the attitudes and behaviors of the patients and the approach of the physician. Physician-related preferences were eliminated because all patients attended the same center. Although previous research has reported that OS provides superior skeletal correction,^15,19^ some patients may refuse surgery. No differences in the initial values were observed between groups in this study, indicating that the patients treated with HA did not have meaningfully different characteristics from patients who preferred OS, who did not demand OS. Therefore, the results of two different treatment methods could be clearly monitored because the patients were similar.

Stereophotogrammetry is very useful in recording the complex 3D morphology and measuring dimensions of the human face in realistic dimensions^36^ and may cause software errors, requires frequent calibration (every 15 days), is expensive, and cannot evaluate hard tissue without X-ray. Various strategies have been employed for the superimposition of consecutive 3D facial images in literature. For instance, Day and Lee^37^ (2006) based their registration solely on the broad surface of the forehead. Soncul and Bamber^38^ (2004) utilized a five-point method involving bilateral En, Ex, and Na landmarks. McCance, et al.^39^(1992), on the other hand, combined the standard five-point approach with additional randomly distributed points across the forehead.

Nevertheless, the precision of this process can be compromised by minor inconsistencies in landmark placement. Specifically, since bilateral En and Ex points tend to lie nearly along the same horizontal line, relying solely on these landmarks may lead to inaccuracies in alignment.^40^ Prior research has also indicated that using only the wide forehead region—especially in individuals with broad and flat forehead contours, as often seen in some Asian populations—can result in lateral deviations of the superimposed images.^40^

To minimize such errors, this study employed a more stable composite registration area, incorporating not only the wide forehead surface but also the nasal bridge and the lateral extensions of the bilateral Ex points, as previously described.^32,41^

In this study, HA was applied with gradual activation, which provides better bone remodeling in the glenoid fossa and condyle, thus showing more skeletal improvement.^42^ To yield a response in functional treatment, progress was started with 4 mm of activation, which is the minimum amount recommended to exceed the minimum tension threshold.^43^ Although stepwise mandibular advancement has been postulated to yield superior skeletal adaptations relative to single-step activation, current evidence remains inconclusive.^29^ While certain investigations have demonstrated enhanced mandibular advancement and attenuated dentoalveolar compensations with gradual activation, other studies report no significant differences in skeletal and dental outcomes between the two approaches, aside from increased proclination and anterior displacement of the mandibular incisors in the stepwise group.^29^ Furthermore, the adjunctive application of extraoral appliances—particularly high-pull headgear—has been proposed to augment vertical control and mitigate undesired dentoalveolar effects.^44^ Collectively, these findings suggest that therapeutic response is likely influenced by a complex interplay of factors, including individual growth patterns, appliance configuration, and activation mechanics.

HA is independent of patient cooperation, and transversal expansion can be performed together with sagittal activation and provide skeletal corrections by increasing the total mandibular length.^45^ The mandibular corpus length increased significantly in this study, however, complications such as crown fracture or separation, screw loosening, rod distortion, pivot fracture, and soft tissue injuries may be found in HA treatment.^14^ Moreover, the device should be used with extreme caution in patients who are not growing, whose skeletal changes are minimal, and treatment effects are limited to the dentoalveolar area. There is also an increased risk for the development of ‘dual bite’ with symptoms of dysfunction from the TMJ as a possible consequence.^18^ Moreover, the changes seen with the use of the HA in adults may simply be the result of “mandibular repositioning”^46^ and the proclination of the lower incisors.^47^

The first use of BSSO procedures for the correction of mandibular deformities was described in 1953.^48^ The most common problem in patients with OS treatment is cranial nerve damage and sensitivity change (during the BSSO, the risk of damage to the inferior alveolar nerve of the mandibular branch of the trigeminal nerve is quite high).^49^ These patients also experience a loss of function, such as drooling, unnoticeable food particles remaining on the lips and/or chin, errors in pronunciation, pain, cheek biting, eating, and kissing.^50^

Temporomandibular joint problems, ranging from intra-articular noise to pain and from limitations in mouth opening to condylar resorption, are the second most common problems of OS.^48^ Bleeding, auditory tube function and hearing problems, infections, and bad split were reported as other possible side effects.^51^ Also, the cost of OS is approximately five to seven times higher than that of orthodontic treatment alone.^51^

A meta-analysis examining the skeletal effect of HA^52^ reported that the SNA angle changed by −0.56 degrees, the SNB angle changed by 1.06 degrees, and the ANB changed by −1.08 degrees. These results are quite close to the relevant values in the HA group in this study. There were no differences when the parameters mentioned were compared with those in the OS group, however, the values in this study were for soft tissues.

Although the meta-analysis revealed that the hard tissue Co-Gn length changed significantly by 1.74 mm, the same authors reported that this small amount may not be clinically significant.^52^ Thus, although the values were like those of the mandibular length and mandibular corpus length in the HA group in this study, these lengths increased significantly in the OS group.

In this study, the anterior facial height increased by 1.5 mm, the posterior facial height increased by 3 mm, and the vertical angle increased by one degree after treatment in the HA group. Although these values differed significantly within the group, they did not differ significantly compared with the changes in the OS group. These results are consistent with the results of the meta-analysis, which reported only a 0.17-degree increase in the mandibular plane angle following HA treatment and that HA had little effect on the mandibular plane angle in the clinic.^52^ Notably, the meta-analysis was conducted on cephalometric studies, whereas this study evaluated soft tissues. The increase in the posterior facial heights in both groups in this study was almost the same as the increase in the S-Go height (3.4 mm) in a cephalometric study.^53^ Many other studies reported that, as well as this study, neither the anterior and posterior facial heights nor the mandibular plane angle (SN-GoMe) were significantly affected.^53^ Conversely, one study reported a decrease in posterior facial height with OS (2 mm), which the authors attributed to remodeling and reformation during post-operative healing.^15^

A study in which only mandibular advancement was evaluated with 3D methods^54^ found that the mentolabial fold tightened after surgery, which is consistent with this study, and the mentolabial angle increased similarly in both groups. This may be attributed to the fact that the lower lip is freed from being trapped between the teeth because of the decrease in overjet with treatment.

In this study, no change in the gonial angle was observed, similar to another study, in which no significant change was noted in the Co-Go-Me angle.^55^ We can conclude that both protocols (HA and OS) can be safely applied in all patients with class II skeletal malocclusion in whom the gonial angle is acceptable (between 128 and 115 degrees^56^).

A cone-beam computed tomography (CBCT) study showed that the location of pogonion in the HA group was significantly different from that in the control group, it was located 1.7 mm anteriorly in the HA group. However, this study was conducted in younger patients and reported short-term results.^57^ Other studies found that the pogonion point was located anteriorly after HA treatment in patients younger than those in this study^53,58^ but no differences were observed when the HA group was compared with the control group. There was a similar result in the HA group in this study, but the pogonion showed a clear average increase in OS of 3 mm more forward in the anteroposterior direction (HA 0.78 mm and OS 3.82 mm). This is very similar to the results of a cephalometric evaluation study that compared the effects of HA and OS (HA: 1.28 mm; OS 4.05 mm).^15^ The same study also observed that the increase in facial convexity angle measured from the soft tissue nasion-subnasale and soft tissue pogonion in the OS group (5.45 degrees) was almost the same as this study (5.10 degrees). The most profound differences between the OS and HA groups were greater mandibular base advancement (mandibular length, corpus length, facial convexity angle, and Pg_z), which led to greater reductions in soft tissue profile convexities. The forward movement of the pogonion indicates that skeletal movements were more active in improving the facial profile and correcting increased overjet in the OS group. In the HA group, we attribute the slight expansion in the alar base width, which we consider clinically insignificant, to the activation of the hyrax screw used for expansion.

The lack of significant differences in baseline cephalometric parameters between the HA and OS groups confirms that the two cohorts were comparable at the outset, thereby strengthening the validity of the treatment effect comparisons. The cephalometric outcomes in this study agree with a previous study, showing greater skeletal advancement in the OS group, particularly in SNB and Pg-NA perpendicular measurements.^15^ As in their findings, data in this study revealed more pronounced mandibular advancement and ANB reduction in OS patients, while HA patients showed more dentoalveolar compensation. Regarding airway dimensions, the CV2′-MPW (sagittal oropharyngeal airway length) increased significantly within both groups, but no significant intergroup difference was detected. This supports previous evidence that both modalities may improve pharyngeal airway space, however, the magnitude of this change may not be solely dependent on the skeletal advancement technique.^29^

Despite the complications and possible risks of OS treatment, many patients are satisfied with the results and recommend OS to those who need it.^59^ Early treatment can also improve oral health and oral health-related quality of life.^51^ We agree that HA is a very reasonable and appropriate option when patients refuse surgery, or their main goal is not to improve their facial aesthetics.^19^

### Limitations

This study has several limitations, including the relatively small sample size, the absence of a control group, the lack of long-term follow-up, and its retrospective design, which may reduce the strength of causal inferences. Furthermore, although a cephalometric skeletal analysis was included to enhance the interpretation of soft tissue changes, the evaluation was limited to 2D lateral cephalometric radiographs, which do not fully capture 3D skeletal dynamics. Three-dimensional hard tissue evaluations using cone-beam computed tomography (CBCT) were not performed, in accordance with the “as low as reasonably achievable” (ALARA) principle. Radiographic imaging was strictly limited to clinically necessary situations, and additional radiation exposure for research purposes was avoided, consistent with internationally recognized ethical standards. This study mainly differed from previous studies in its evaluation of soft tissues. Although this approach is in line with today’s aesthetic concept approach, it also has limitations, such as soft tissue thickness, muscle structures, and biological and individual differences.

## Conclusions

Despite the limitations of the study, several key conclusions can be drawn. Patients in the HA and OS groups achieved class I occlusal relationships and reductions in the convexity of the soft tissues over the treatment period (T1-T0). However, more skeletal contribution was observed in the OS group than in the HA group because of the increase in mandibular length and mandibular corpus length. The only significant 3D soft tissue coordinate change between the groups was the anterior displacement of the pogonion in the sagittal plane, which was further corroborated by cephalometric findings showing significant increases in SNB angle and Pg-NA perpendicular measurements in the OS group. This anterior movement of the pogonion contributed to a reduction in soft tissue convexity, as reflected by the increased facial convexity angle. A clinically insignificant increase in alar base width was observed in the HA group. Additionally, both treatment modalities resulted in significant improvements in sagittal oropharyngeal airway length, without significant intergroup difference.
